# Impact of Admission Glycosylated Hemoglobin A1c on Angiographic Characteristics and Short Term Clinical Outcomes of Nondiabetic Patients with Acute ST-Segment Elevation Myocardial Infarction

**DOI:** 10.1155/2015/274892

**Published:** 2015-12-01

**Authors:** Islam El-sherbiny, Baher Nabil, Tamer Saber, Fathy Elsayed Abdelgawad

**Affiliations:** ^1^Cardiology Department, Faculty of Medicine, Zagazig University, Zagazig 44519, Egypt; ^2^Internal Medicine Department, Faculty of Medicine, Zagazig University, Zagazig 44519, Egypt; ^3^Medical Biochemistry Department, Faculty of Medicine, Al-Azhar University, Cairo 11651, Egypt

## Abstract

We aimed to assess the predictive value of admission HbA1c level in nondiabetic patients presented by acute STEMI, on outcome of PCI and short term outcome of adverse cardiac events.* Methods*. 60 nondiabetic patients were admitted to Cardiology Department, Zagazig University Hospital, with acute STMI: 27 patients with HbA1c levels of 4.5% to 6.4% (group 1), 17 patients with HbA1c levels of 6.5% to 8.5% (group 2), and 16 patients with HbA1c levels higher than 8.5% (group 3). Either invasive intervention was done at admission by (pPCI) or coronary angiography was done within month (3–28 days) from taking thrombolytic. Participants were followed up for 6 months.* Results*. There was significant difference among different groups of HbA1c as regards the number of diseased vessels, severity of CAD lesions (*p* value < 0.01), and TIMI flow grades (*p* value < 0.05). There was significant difference among different groups as regards the adverse cardiac events on short term follow-up period (*p* value < 0.05).* Conclusion*. The present study showed that admission higher HbA1c level in patients presented by acute STEMI is associated with more severe CAD, lower rate of complete revascularization, and higher incidence of adverse cardiac events.

## 1. Introduction

Stress hyperglycemia in ST-segment elevation myocardial infarction (STEMI) patients was found to be associated with significantly increased rates of mortality and congestive heart failure and shock [1–3]. Most of these studies, however, were in trials of fibrinolytic therapy; conversely the evidence linking hyperglycemia with an adverse prognosis in patients treated with primary percutaneous coronary intervention (pPCI) is limited and derived mainly from observational registries [4].

In acute myocardial infraction (AMI), stress hyperglycemia commonly occurs secondary to increased catecholamine levels, so looking only at plasma glucose levels at the time of an AMI cannot predict the prognosis [5]. Glycosylated hemoglobin A1c (HbA1c) is a measure of the average blood glucose levels over 2 months [6] and is minimally affected by acute hyperglycemia often observed in myocardial infarction (MI).

Elevated HbA1c is an important determinant of atherosclerosis beyond the risk associated with established diabetes [7].

*Aim of the Work*. Our study aimed to assess the predictive value of admission HbA1c level in nondiabetic patients presented to Zagazig University Hospital by acute STEMI, on outcome of pPCI and short term outcome of adverse cardiac events.

## 2. Patients and Method

We performed a single-center, observational prospective study of sixty nondiabetes patients with acute STEMI, candidate for reperfusion therapy admitted to Cardiology Department, Zagazig University Hospital, between January 2013 and December 2013.

### 2.1. Inclusion Criteria

Confirmed admission diagnosis of acute STEMI, within 12 h of symptom onset and with persistent ST-segment elevation or new or presumed new LBBB, early mechanical pPCI, or pharmacological reperfusion should be performed as early as possible.

### 2.2. Exclusion Criteria

Patients with passed time MI, patients who have undergone rescue PCI, patients who have contraindication to dye as dye allergy and chronic kidney disease, patients with liver cell failure, haemoglobinopathies, haemolytic anaemia, chronic malaria, or severe iron deficiency anaemia due to giving unreliable results leading to inaccurate HbA1c level, and patients with past history of diabetes or on antidiabetic treatment were excluded.

### 2.3. Clinical Data and Blood Samples

Main demographic data and the five major risk factors obesity, history of hypertension, dyslipidemia, family history, and cigarette smoking were carefully evaluated.

HbA1c levels of all patients were measured within 3 hours of admission, using Siemens DCA analyzer for quantitative assay for HbA1c in blood. Both the concentrations of HbA1c specifically and the concentration of total hemoglobin are measured and the ratio reported as percent HbA1c. All of the reagents for performing both reactions are contained in the DCA HbA1c cartridge [8] and using a method which is National Glycohemoglobin Standardization Program (NGSP) [9].

Patients were divided into three groups according to the level of HbA1c with cut-off 6.5% as a diagnostic criteria of diabetes mellitus according to (2010) ADA Diagnosis and Classification of Diabetes Mellitus [10]: 27 patients with HbA1c levels of 4.5% to 6.4% (group 1), 17 patients with HbA1c levels of 6.5% to 8.5% (group 2), and 16 patients with HbA1c levels higher than 8.5% (group 3).

### 2.4. Angiography

Either invasive intervention was done at admission by pPCI or coronary angiography was done within month (3–28 days) from taking thrombolytic in stable asymptomatic patients according to the best evidence based medicine [11, 12]. 45 cases were subjected to pPCI and 15 cases received thrombolytic. For pPCI wiring culprit artery based upon ECG, revascularization of culprit artery only was done unless patient is in cardiogenic shock [13]. We used the visual method for evaluation and assessment of number of significantly diseased coronary arteries, with special focus on infracted related artery (guided by ECG) on type of the lesion either type A, B, or C [14]. Also, we used TIMI flow grading system in pPCI cases [15]. Angiographic results were interpreted by two angiographers who were blinded to clinical or demographic data. The study protocol was approved by the institutional ethics committee and all patients provided written, informed consent before study entry.

### 2.5. Follow-Up Period

Follow-up for six months after the onset of MI mainly for (1) heart failure, (2) reinfarction, and (3) mortality was through phone calls followed by hospital visit in outpatient clinic.

### 2.6. Statistical Analysis

Statistical presentation and analysis of the present study were conducted, using the mean, standard deviation, analysis of variance (ANOVA) test, and chi-square test by SPSS V 21, Student's *t*-test was used to test the significance of the difference between two independent sample means; value of <0.05 indicates significant results.

## 3. Results

Baseline clinical characteristics and angiographic data regarding number of diseased vessels and lesion type are shown in Table 1. There was no significant difference between the three groups regarding age, sex, hypertension, smoking, family history, and obesity (*p* value > 0.05).

**Figure 1:**
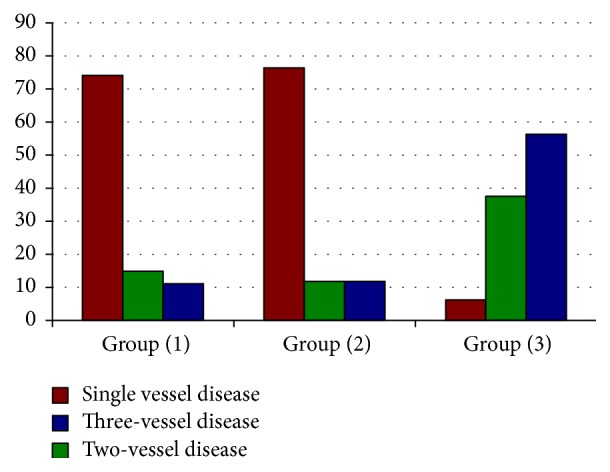
Number of diseased vessels in the study groups (1, 2, and 3).

Regarding number of diseased vessels, there was high statistically significant difference between the three groups (*p* value < 0.001), Figure 1.

**Figure 2:**
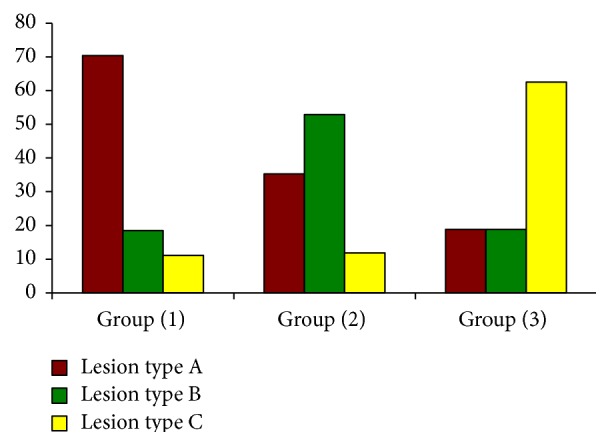
Lesion type in relation to the study groups according to HbA1c.

There was high statistically significant difference between the three groups regarding lesion types A, B, and C (*p* value 0.000), Figure 2.

**Figure 3:**
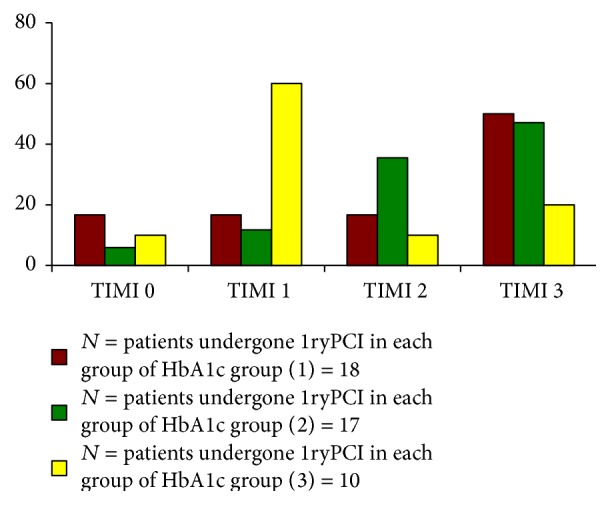
TIMI flow grade in primary PCI cases (*n* = 45).

Table 2 shows significant difference among different groups of HbA1c as regards the degree of successful reperfusion. This was supported by the significant difference among different groups as regards TIMI flow grades with higher percentage of TIMI grade 3 in group 1 and higher percentage of TIMI grade 1 in favor of group 3 (*p* value < 0.05), Figure 3. Also as shown in Table 2 patients in group 1 have higher rates of TIMI flow grade 0 (16.7%) as compared to group 2 (5.9%) and group 3 (10%).

**Figure 4:**
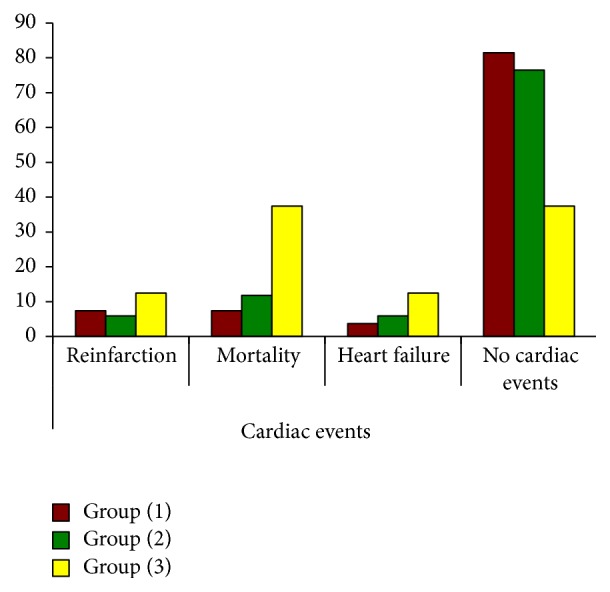
Adverse cardiac events in the study groups (1, 2, and 3).

Adverse cardiac events on short term follow-up period were shown in Table 3; statistically significant difference among different groups of HbA1c with higher mortality, reinfarction, and heart failure in group 3 (*p* value < 0.05) is shown in Figure 4. A multiregression analysis in the present study confirmed that HbA1c over 6.5% is an independent predictor outcome of adverse cardiac events.

## 4. Discussion

In acute myocardial infraction (AMI), stress hyperglycemia commonly occurs secondary to increased catecholamine levels, so looking only at plasma glucose levels at the time of an AMI cannot predict the prognosis [5]. Glycosylated hemoglobin A1c (HbA1c) is a measure of the average blood glucose levels over 2 months [6] and is minimally affected by acute hyperglycemia often observed in myocardial infarction (MI).

In the present study there was significant difference among different HbA1c groups as regards the number of diseased coronary vessels with higher percentage of three-vessel disease, better than higher number of three-vessel disease in group (3). This was concordant with previous studies by Cakmak et al. [16] and Kassaian et al. [17]. This can be explained by insulin resistance in hyperglycemia promoting molecular mechanism by Advanced Glycation End Products (AGEs) which are intimately involved in the pathophysiology of cardiovascular disease by stimulating inflammation, contributing to atheroma formation modulating vascular stiffness and the disturbed endothelial function by reduction of nitric oxide release and increased vascular smooth muscle proliferation [18] and increase of HbA1c one percent is associated with 2.8-fold increase in CAD and in severity of coronary artery lesions, beyond high risk profile of those patients; it is worth mentioning that even HbA1c value in normal range is associated with presence and severity of CAD [19].

On the contrary, this was discordant with Ertem et al. [20] who found no significant difference between HbA1c level and severity of CAD. This discrepancy may be due to the different score for assessment of the severity CAD in their study or the sample size or population difference.

In the present study there was significant difference among different HbA1c groups as regards lesion type (A, B, and C) with higher lesion type A in group (1) and lesion type C in group (3). This was in agreement with Kassaian et al. [17] who found highly significant difference among HbA1c groups as regards lesion type C. This may be explained by the fact that diabetes is associated with more extensive coronary artery lesion, more complex lesion, more diffuse excessive tortuosity in the proximal segment, extreme angulations, and total occlusion [20].

In the present study there was significant difference among different HbA1c groups as regards TIMI flow grades in patients who had undergone pPCI, with higher percentage of TIMI 3 in group (1) and significant higher percentage of TIMI 1 in favor of group (3) and this was in agreement with Planner et al. [4]. This finding may be explained by the fact that hyperglycemia is associated with higher rate of TIMI 0\1 and lower rate of complete revascularization TIMI and hyperglycemia adversely affect platelets function and endothelial function, promote inflammation, and result in procoagulable condition; it is worth mentioning that hyperglycemia per se in STEMI leads to impaired coronary flow on presentation and after pPCI [4]. This goes with our finding that TIMI 3 complete revascularization is higher in group (1), the least level of HbA1c, and TIMI 1 flow has statistical significant difference between groups (1) and (3) with higher percentage in favour of group (3), the highest level of HbA1c, beyond the fact that patients with higher HbA1c on admission have larger myocardial infarction and less frequently have open infracted artery.

In the present study we assess HbA1c level on the short term outcome (six months) to detect major adverse cardiac event as mortality, heart failure, and reinfarction. In the present study there was significant difference among different HbA1c groups as regards adverse cardiac events; this was concordant with Cakmak et al. [16] and Kassaian et al. [17]. This may be explained by the fact that higher HbA1c level at admission was associated with worse cardiometabolic characteristics, larger infarct size, more extensive coronary artery lesion, higher percentage of TMI 1 flow, and lower rate of complete revascularization TIMI 3 flow.

## 5. Conclusion 

The present study showed that admission higher HbA1c level in nondiabetic patients presented by acute STEMI is associated with more severe CAD, lower rate of complete revascularization TIMI 3, and higher incidence of adverse cardiac events and mortality. Higher HbA1c level may be considered for risk stratification of patients presented by acute STEMI who are amenable to primary PCI, but these findings need to be verified by multicenter and larger cohort studies. In addition, this study was limited to Egyptian subjects, so conclusions for other ethnic groups are needed.

## Figures and Tables

**Table 1:** Baseline clinical and angiographic data of patients according to HbA1c.

Level of glycosylated hemoglobin				
	Group (1) <6.5% (*n* = 27)	Group (2) 6.5%–8.5% (*n* = 17)	Group (3) >8.5% (*n* = 16)	*p* value
Age, years (mean ± SD)	57.9 ± 10.2	58.3 ± 7.1	57.3 ± 8.5	NS
Sex, *n* (male/female)	23/4	13/4	12/4	NS
Smoking, %	55.6%	47.1%	37.5%	NS
Hypertension, %	48.1%	47.1%	68.8%	NS
Obesity (BMI > 30 kg/m^2^), %	22.2%	17.6%	25%	NS
Family history	11.1%	11.7%	18.8%	NS

Angiography (number of diseased vessels), *n* (%)				
Single vessel	20 (74.1%)	13 (76.4%)	1 (6.2%)	0.000
Double vessel	4 (14.8%)	2 (11.8%)	6 (37.5%)	0.12
Triple vessel	3 (11.1%)	2 (11.8%)	9 (56.3%)	0.001

Lesion type, *n* (%)				
A	19 (70.4%)	6 (35.3%)	3 (18.8%)	0.000
B	5 (18.5%)	9 (52.9%)	3 (18.8)	
C	3 (11.1%)	2 (11.8%)	10 (62.5%)	

**Table 2:** TIMI flow grade in primary PCI cases (*n* = 45).

	TIMI 0	TIMI 1	TIMI 2	TIMI 3	Sig.
Group (1) (*N* = 18)	(3) 16.7%	(3) 16.7%	(3) 16.7%	(9) 50%	
Group (2) (*N* = 17)	(1) 5.9%	(2) 11.8%	(6) 35.5%	(8) 47.1%	0.04
Group (3) (*N* = 10)	(1) 10%	(6) 60%	(1) 10%	(2) 20%	

**Table 3:** Adverse cardiac events in the study groups according to HbA1c.

Level of glycosylated hemoglobin				
Adverse cardiac events (NO) %	Group (1) <6.5% (*n* = 27)	Group (2) 6.5%–8.5% (*n* = 17)	Group (3) >8.5% (*n* = 16)	*p* value
Reinfarction	(2)	(1)	(2)	0.04
	7.4%	5.9%	12.5%	
Mortality	(2)	(2)	(6)	
	7.4%	11.8%	37.5%	
Heart failure	(1)	(1)	(2)	
	3.7%	5.9%	12.5%	
No cardiac events	(22)	(13)	(6)	
	81.5%	76.5%	37.5%	
